# Reverse Obliquity Intertrochanteric Hip Fractures: A Technique Guide for Using a Blocking Screw to Enhance Fixation

**DOI:** 10.7759/cureus.117493

**Published:** 2026-10-05

**Authors:** Nathan Forren, Daniel Sabo, Brent Whitehead, Vincenzo Gentile, Adam Hoffman, Tyson Schrickel, John V Gentile

**Affiliations:** 1 Department of Orthopedic Surgery, St. Elizabeth Youngstown Hospital, Youngstown, USA; 2 Department of Medicine, Heritage College of Osteopathic Medicine, Ohio University, Warrensville Heights, USA; 3 Department of Orthopedic Trauma, St. Elizabeth Youngstown Hospital, Youngstown, USA

**Keywords:** blocking screw, femur intertrochanteric fracture, reverse oblique, surgical technique guide, technical report

## Abstract

Reverse oblique (RO) intertrochanteric (IT) femur fractures are an unstable fracture variant because of loss of lateral-wall support, making cephalomedullary nail (CMN) fixation the preferred implant strategy for these fractures. Despite the biomechanical advantages of CMNs, lateral migration of the nail within the canal may permit fracture shear during weight-bearing and contribute to loss of reduction or fixation failure. The technique discussed in this article describes using a blocking screw in RO IT hip fractures to limit potential lateralization of the nail in the distal aspect of the proximal femoral canal and neutralize this shear tendency. The screw is placed adjacent to the intramedullary nail to support maintenance of reduction and alignment. This technical guide describes the operative steps and considerations for selective use of the screw; it does not present clinical or biomechanical outcome data. Further study is needed to determine its effect on fracture healing and fixation stability.

## Introduction

Intertrochanteric (IT) hip fractures are common as the population continues to age and are associated with significant morbidity and mortality. These fractures can be particularly challenging when certain patterns are present, such as the reverse oblique (RO) fracture pattern. RO fractures are unstable and are more prone to failure with standard fixation techniques [1,2]. Although cephalomedullary nails (CMNs) are commonly used for RO IT fractures [3-5], there remains a risk of nail migration within the canal, which may allow fracture shear during weight-bearing and contribute to failure [1]. In these cases, achieving and maintaining fracture reduction are critical. The unique geometry of RO fractures may allow the proximal fragment to displace laterally relative to the distal femoral shaft, contributing to instability and risk of fixation failure. This technique guide describes using a blocking screw placed lateral to the intramedullary nail and distal to the fracture site as an adjunct to standard CMN fixation.

The goal of the described technique is to provide additional mechanical support after appropriate nail selection and fracture reduction have been achieved. The blocking screw is intended to act as a lateral-wall substitute by limiting potential lateral migration of the intramedullary nail within the femoral canal. The screw is placed in cancellous bone proximal to the isthmus, lateral to the nail, and distal to the fracture. By supporting alignment and reduction, this approach may help reduce the tendency toward lateral nail translation. The proposed benefit is theoretical; this technical guide does not establish improved healing, union rates, or clinical outcomes.

## Technical report

As mentioned, intramedullary fixation of an RO fracture (Figure 1) may be prone to failure because of the unique geometry of the pattern. Figures 2, 3 demonstrate lateral migration of the intramedullary nail within the femoral canal and associated malreduction. The technique described provides an additional mechanical adjunct intended to limit this potential migration. This manuscript is a technical guide based on operative experience and does not report a case series or outcome study. The Institutional Review Board at the authors’ institution reviewed this project and determined that it did not meet the criteria for human-subject research.

**Figure 1:**
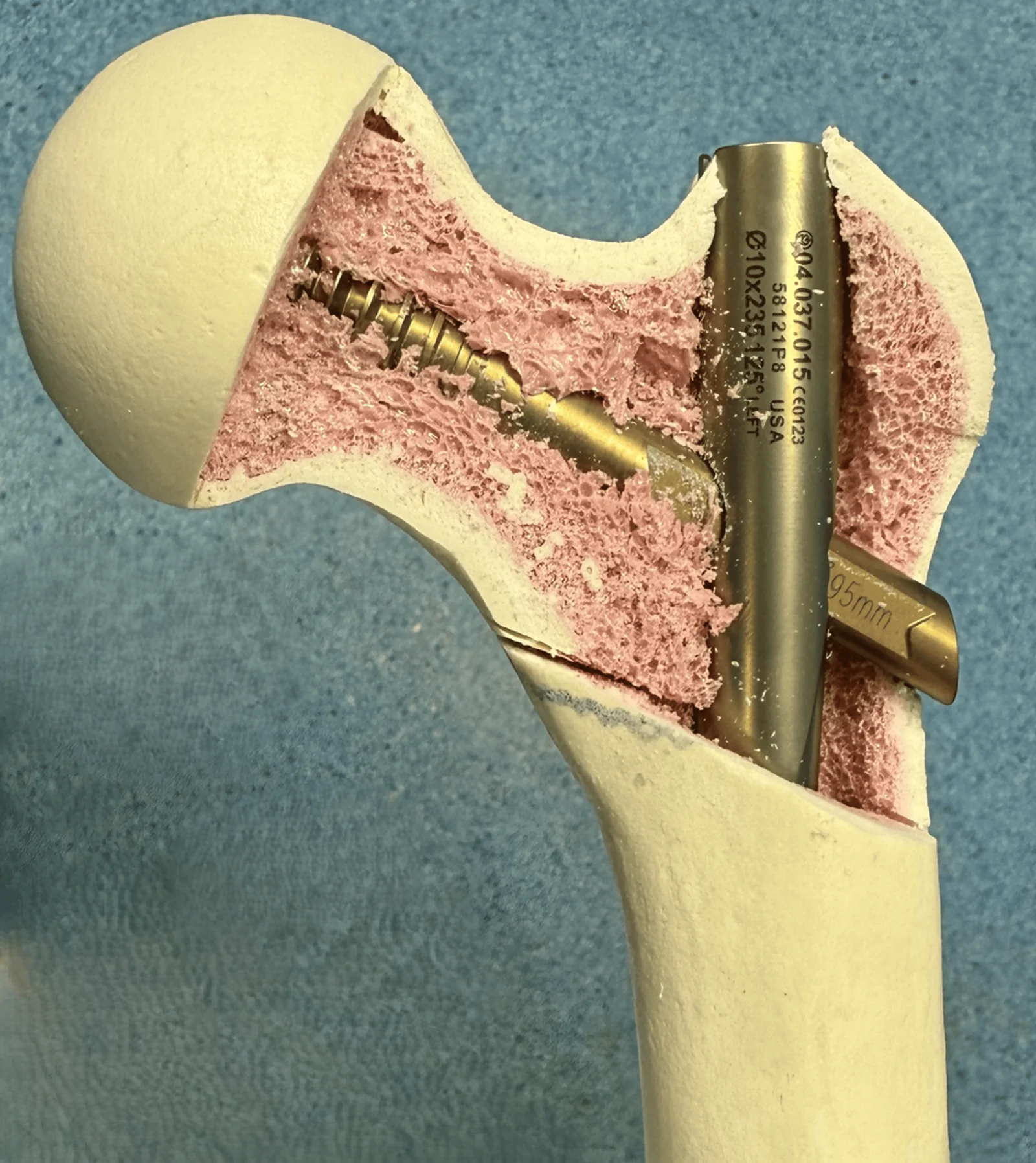
Typical complete nail fixation with a short cephalomedullary nail

**Figure 2:**
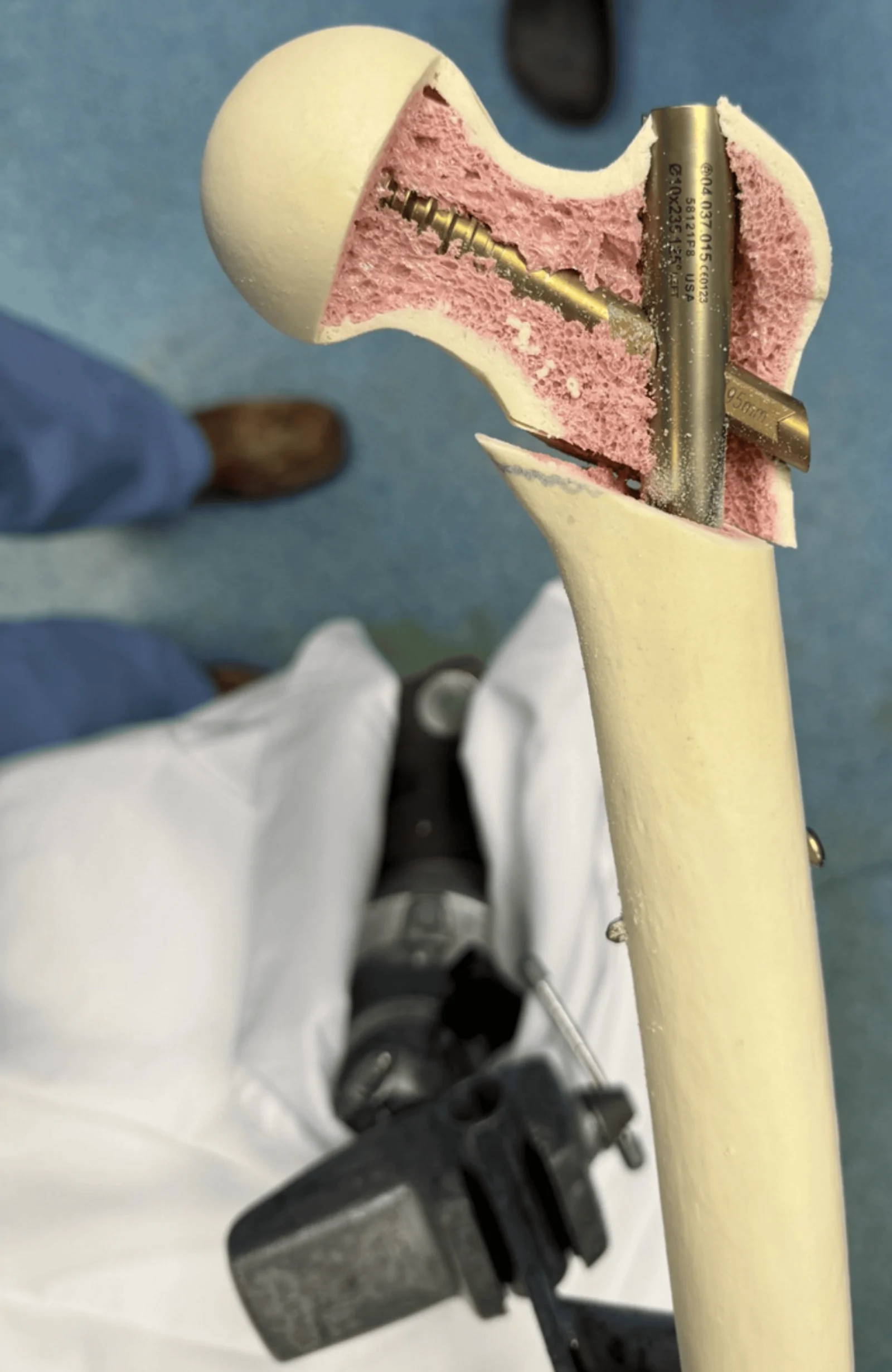
Soft cancellous bone laterally to the nail, proximal to the isthmus, with lateralization of the nail and malreduction

**Figure 3:**
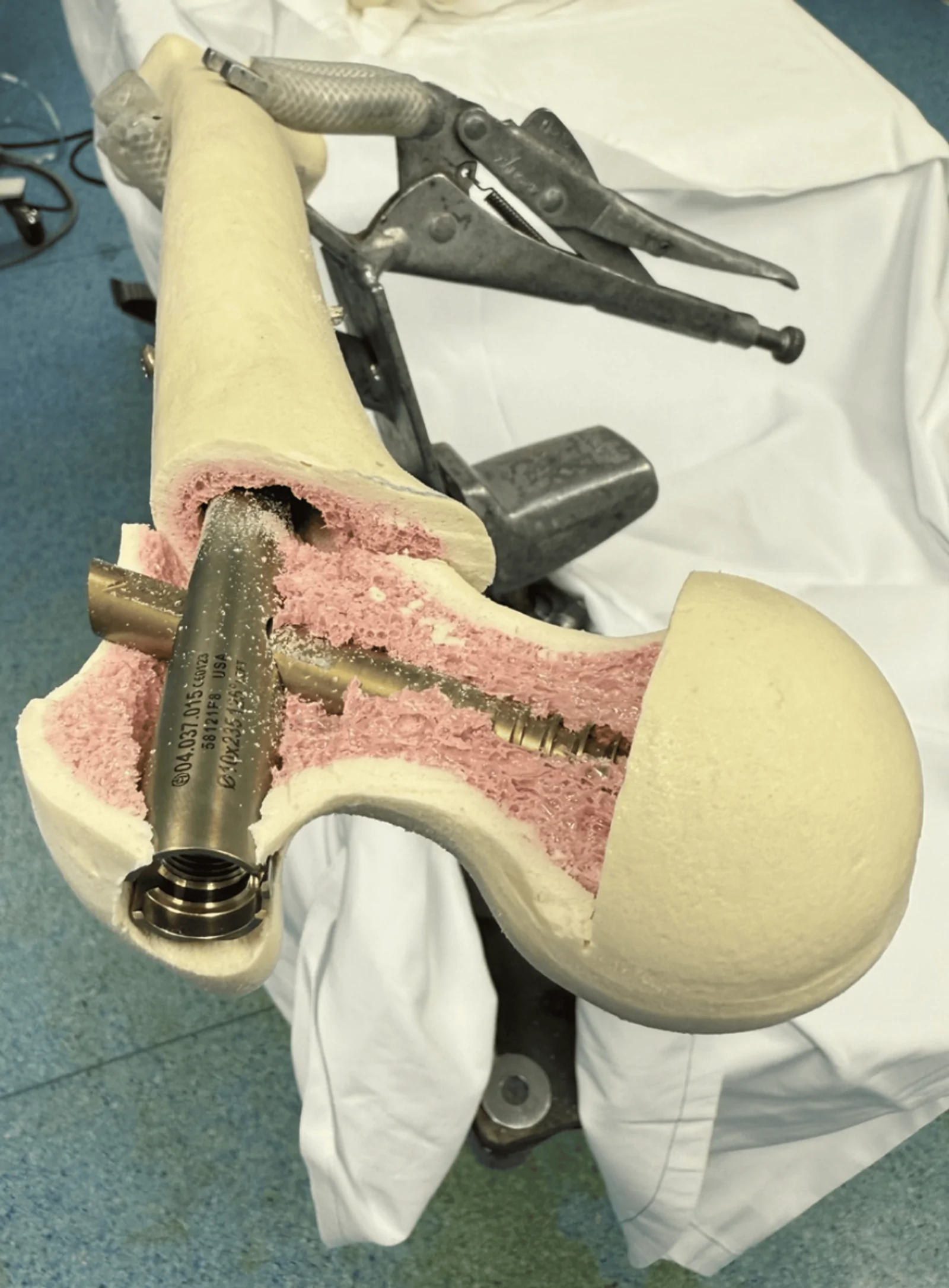
Soft cancellous bone laterally to the nail, proximal to the isthmus, with lateralization of the nail and malreduction

Novel technique

The patient is anesthetized under the care of the anesthesia team, and fracture boots are placed on both lower extremities. The patient is then transferred to the Hana fracture table. Both lower extremities are placed in the holders, and the patient’s operative arm is placed across the body to aid access to the surgical site. Fluoroscopy is used to examine the fracture radiographically. Using the Hana table, the fracture is reduced with manual manipulation and confirmed fluoroscopically. The femoral neck angle is confirmed, and canal diameter is approximated. For this technique, the DePuy Synthes (Raynham, MA) TFN-ADVANCED™ Proximal Femoral Nailing System is used.

The patient is prepped and draped in standard sterile fashion, and fluoroscopy is brought back in. The nail entry point is identified, and a guidewire is inserted at the tip of the greater trochanter on an anteroposterior (AP) radiographic view and at the center of the greater trochanter on the lateral view. The proximal femur is accessed using an opening reamer, and the canal is reamed as appropriate based on selection of a long or short CMN. If using a short CMN as described in this technique, the nail is inserted, and the aiming arm is attached as appropriate for the femoral neck angle. The guidewire is inserted into the femoral head, centered on AP and lateral hip views, to determine lag-screw length. The guide sleeve is inserted, and the femoral neck and head are drilled as appropriate. The screw or blade is then inserted into the femoral head and locked into the CMN.

The use of an undersized nail is not the intended indication for this technique, as blocking screws are not intended to compensate for inadequate nail selection. Nail diameter and length should first be selected using standard preoperative templating and intraoperative assessment of canal dimensions. Satisfactory fracture reduction and CMN fixation should be achieved before considering placement of a blocking screw. The blocking screw may be considered when fluoroscopy demonstrates residual lateral metaphyseal space adjacent to an appropriately sized nail, particularly at the level of the distal taper of the larger proximal nail diameter and proximal to the isthmus. Use of the blocking screw is patient-selective. There must be adequate lateral cortical bone stock and sufficient space to permit safe bicortical screw placement adjacent to the nail. A blocking screw should not be used when the nail already occupies most of the proximal femoral metaphyseal canal or when the lateral cortex is fractured at the proposed screw-placement level. It is also important to acknowledge that you should not risk loss of reduction for placement of the blocking screw, nor should you risk lateral wall blowout of the femoral cortex if the surgeon feels the lateral wall is thin.

When the selection criteria are met, the goal is for the blocking screw to be placed at the level of the distal taper of the larger proximal nail diameter along the lateral aspect of the nail, where the canal diameter may be largest and most amenable to screw placement. Using fluoroscopy, identify the femoral shaft just lateral to the nail and at the level of the proximal nail diameter; sharply incise the skin with a scalpel and bluntly dissect to the proximal femoral cortex; confirm the appropriate drill-bit position fluoroscopically, and drill anterior to posterior through the femoral canal just lateral to the nail; and measure and place the lateral blocking screw (Figure 4).

**Figure 4:**
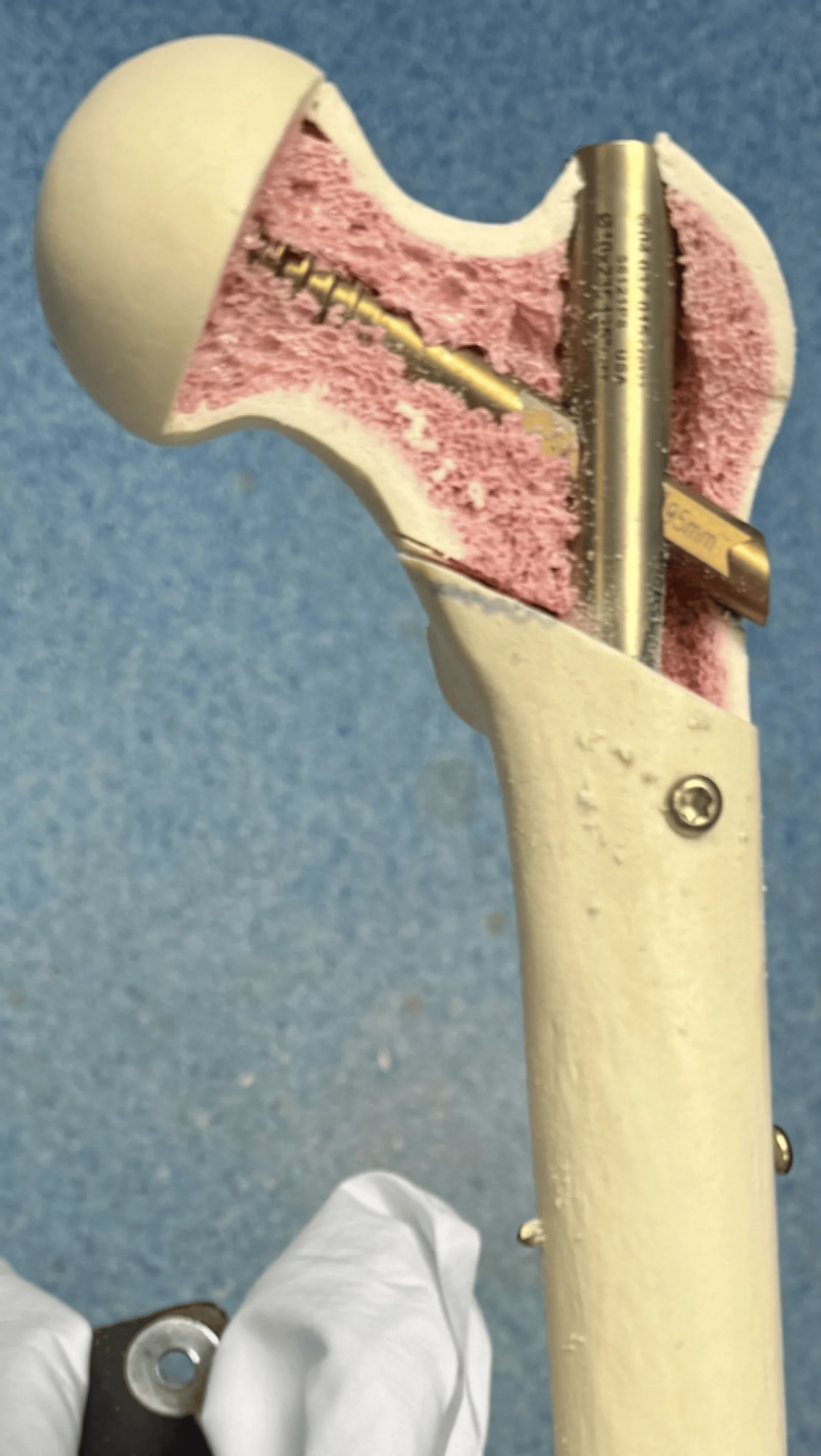
Lateral blocking-screw placement adjacent to the nail with improved reduction

With this technique, it is important to identify the diameter of the proximal femur in the metaphyseal region relative to the isthmus. If the selected nail diameter occupies most of the proximal femoral metaphysis, the ability to place a blocking screw bicortically is impaired. Overall, the quality of the reduction should not be sacrificed to place the blocking screw.

## Discussion

The use of blocking screws in cephalomedullary nailing remains an area of interest in our orthopedic surgery program. We are particularly focused on their potential role in limiting lateralization of the CMN within the distal aspect of the proximal femoral canal. As mentioned previously, orthopedic surgeons continue to encounter instability associated with RO IT fractures [1,2]. The current standard treatment is cephalomedullary nailing with reduction. This, however, may not fully prevent lateralization of the proximal fracture block. The blocking screw technique is intended as an adjunct to support reduction in selected cases. Because this manuscript is a technical guide, it does not provide clinical or radiographic outcome data establishing that the technique prevents lateralization or improves outcomes.

Using a blocking screw for RO IT fractures may offer an option that does not rely exclusively on the cephalomedullary device. One published article examining fixation of RO IT fractures describes using mini-fragment plates and cerclage wires to support lateral translation of the femur. This requires soft-tissue stripping to access the femur and may add time to the case [6]. In contrast, the described technique uses a small percutaneous incision and a fluoroscopically guided screw trajectory. Although we believe this approach may be less invasive, this technical guide does not compare operative time, tissue devitalization, fracture healing, or clinical outcomes with plate or cerclage techniques. The small incision, fluoroscopic marking, and drilling using the same principles as “perfect circles” are familiar concepts for orthopedic surgeons performing distal interlocking screw placement in long CMNs. Body habitus, including extreme obesity, or prior surgery to the anterior proximal thigh may complicate the technique.

Compared with more soft-tissue-invasive procedures, including cerclage wires and mini-fragment plates [6], the blocking screw technique uses a small incision on the anterior aspect of the thigh under fluoroscopic guidance. This allows the clinician to make a percutaneous skin incision and minimizes soft-tissue stripping around the bone. The drill bit is advanced with oscillation to limit soft-tissue injury.

The blocking screw technique may be integrated into the case when the described anatomic criteria are met. Potential complications include sensory nerve injury involving the lateral femoral cutaneous nerve and lateral cortical violation or blowout during drilling or screw insertion. These complications have not been systematically evaluated in this technical guide. A practical challenge may be drilling anterior to posterior under fluoroscopy because the C-arm can interfere with drill position. This can be addressed by lowering the fracture table or raising the C-arm, when possible. In patients with a smaller thigh circumference, a shorter drill bit may facilitate positioning under the image intensifier. Future research may include case series, retrospective studies, and biomechanical evaluation once adequate follow-up data are available.

## Conclusions

Reverse-obliquity IT femur fractures remain challenging to treat because of their inherent instability and tendency toward lateral translation despite CMN fixation. The addition of a lateral blocking screw placed adjacent to the intramedullary nail may provide a simple adjunct to standard CMN in selected cases. The technique is intended to limit potential lateral migration of the nail within the proximal femoral canal and thereby support maintenance of fracture reduction and alignment. Appropriate patient selection based on proximal femoral anatomy is important, as adequate space and lateral cortical bone stock must be available for safe bicortical screw placement. Fracture reduction should not be compromised to accommodate the blocking screw. This technical guide does not establish clinical effectiveness, prevention of failure, or improved fracture healing; further biomechanical and clinical studies are necessary to evaluate these outcomes.
